# Experimental Validation of a Microwave Imaging Method for Shallow Buried Target Detection by Under-Sampled Data and a Non-Cooperative Source

**DOI:** 10.3390/s21155148

**Published:** 2021-07-29

**Authors:** Adriana Brancaccio, Giovanni Leone, Rocco Pierri, Raffaele Solimene

**Affiliations:** 1 Dipartimento di Ingegneria, Università degli Studi della Campania Luigi Vanvitelli, 81031 Aversa, Italy; giovanni.leone@unicampania.it (G.L.); rocco.pierri@unicampania.it (R.P.); raffaele.solimene@unicampania.it (R.S.); 2 Consorzio Nazionale Interuniversitario per le Telecomunicazioni-CNIT, 43124 Parma, Italy; 3 Department of Electrical Engineering, Indian Institute of Technology, Chennai 600036, India

**Keywords:** radar imaging, target detection, experimental measurements, microwave imaging

## Abstract

In microwave imaging, it is often of interest to inspect electrically large spatial regions. In these cases, data must be collected over a great deal of measurement points which entails long measurement time and/or costly, and often unfeasible, measurement configurations. In order to counteract such drawbacks, we have recently introduced a microwave imaging algorithm that looks for the scattering targets in terms of equivalent surface currents supported over a given reference plane. While this method is suited to detect shallowly buried targets, it allows one to independently process all frequency data, and hence the source and the receivers do not need to be synchronized. Moreover, spatial data can be reduced to a large extent, without any aliasing artifacts, by properly combining single-frequency reconstructions. In this paper, we validate such an approach by experimental measurements. In particular, the experimental test site consists of a sand box in open air where metallic plate targets are shallowly buried a (few cm) under the air/soil interface. The investigated region is illuminated by a fixed transmitting horn antenna, whereas the scattered field is collected over a planar measurement aperture at a fixed height from the air-sand interface. The transmitter and the receiver share only the working frequency information. Experimental results confirm the feasibility of the method.

## 1. Introduction

Microwave imaging, and in general radar imaging, is a mature research field that finds application in a number of different contexts where pursuing non-destructive investigation is convenient or mandatory [1,2,3,4,5,6,7,8,9,10,11].

Ground Penetrating Radar (GPR) is a radar system that is properly conceived to address non-destructive imaging. Generally, GPRs work in contact with the interface between the air and the medium under investigation. However, there is great interest in achieving target detection through non-contact measurement layouts, for example, with GPRs mounted on a flying platform [12,13]. Indeed, stand-off distance configurations allow for the investigation of regions that are not easily (or safely) accessible, as it happens, for instance, when one has to deal with mine or unexploded device detection [14]. Moreover, a flying GPR can allow for inspecting large areas quickly [15,16].

In this framework, however, the system cost and the achievable performance must be traded-off [17]. This requires finding a compromise between the time needed to collect data, the number of sensors to be simultaneously deployed, and the way transmitter and receivers “communicate”. In this regard, a single-view/multistatic configuration seems convenient, since only one sensor transmits and the others act as mere passive receivers, hence with reduced weight and cost. However, synchronization between TX and RXs is a critical issue when they are mounted on different platforms, since, differently from multi-monostatic arrangement, TX and RXs are no more co-located and hence do not share the same electronic system. In addition, the number of measurement points is directly linked to the number of flying platforms and hence must be reduced as much as possible.

Actually, a number of different processing schemes have been proposed in the literature to address subsurface imaging [18]. Recently, we have introduced a reconstruction scheme that allows one to mitigate the previously mentioned drawbacks [19,20]. This method relies on a certain scattering formulation where, according to the equivalence principle [21], the scattered field is modeled as being radiated by equivalent surface currents that are supported over the air/soil interface, or at some other reference plane whose depth is chosen. A related method that uses equivalence principles for target shape reconstruction is reported in [22], where, however, the multiple experiments arise from using different illuminations (i.e., a multistatic/multiview configuration is employed).

In our approach, basically, the main idea is that if the reference plane is close to the scattering target, then the spatial support of the surface current gives information concerning the transverse location of the target. If it is a priori known that the targets are in close proximity to the air/soil interface, e.g., for detection of a mine [23] or unexploded improvised device [24] (where the targets are often very shallowly buried just to hide from sight), then the reference plane can be set just at the air/soil interface. In this case, the surface currents radiate in free-space, and the related simple Green function can be considered as a propagator. The reconstruction is cast as a 2D inverse problems, since only the targets detection and their transverse locations are looked for, and hence single-frequency data can be employed. Accordingly, RXs do not need information about the TX, except the working frequency. Hence, the source can be considered as being non-cooperative (it does not share information with the receivers). However, it is not opportunistic as in most passive radar, since it is deliberately deployed in the scene. Multi-frequency data can be processed separately (i.e., incoherently) and then combined to counteract aliasing artifacts that can arise if the spatial measurement points are reduced (not properly sampled) [20]. Finally, depth can be explored by performing the reconstructions at different reference planes.

In previous contributions (see [19,20]), we have shown the feasibility of the method by employing synthetic numerical data and some measured data collected under lab conditions for a free-space scattering scenario. However, the method still need to be validated in a realistic scattering scenario.

In this contribution, we aim at pursuing such a task. To this end, the method and the related achievable performances are checked for a more realistic scattering scenario where the background medium is actually not homogeneous. Indeed, the test site mimics a realistic on-field situation, as it consists of an open-air sand box. As to the RXs and the Txs, they are not mounted on flying platforms. However, they are at a stand-off distance from the air/soil interface, and the TX signal is unknown to the RXs but the working frequency is known.

In sum, the advancements that we are conveying in this contribution concern:

- The generalization of the scattering model for a near-field configuration (previous results refer to a far-field case);
- The experimental validation of the approach for a realistic scattering scenario.

The rest of the paper is organized as follows. In Section 2 the scattering model is generalized to deal with the new scattering configuration, whereas in Section 3, the related reconstruction algorithm is briefly described. In Section 4, the experimental set-up as well as a few experimental results are presented and discussed. Conclusions end the paper.

## 2. Scattering Model

In this section, we introduce the adopted scattering model and the necessary notation that is required in the following. A detailed derivation of the model can be found in [19]. Here, we adapt that derivation to the configuration used in the experimental set up (described later).

The background medium is two-layered with the upper half-space being air, while the lower one models the soil. The two half-spaces are separated by a planar interface (i.e., the air/soil interface) located at z=0. The scattering targets are located in the lower half-space (i.e., for z<0) and buried in close proximity to the separation interface. Moreover, the “transverse” investigation domain (i.e., the spatial region where the targets can belong to) is denoted as $D=\left[-x_{M},x_{M}\right]\times \left[-y_{M},y_{M}\right]$. The 2D investigation domain is considered at a fixed depth $z_{T}$. Generally, we will consider $z_{T}=0$ (which is just at the separation interface). Reconstructions at different $z_{T}<0$ can be considered as well in order to explore the depth.

**Figure 1:**
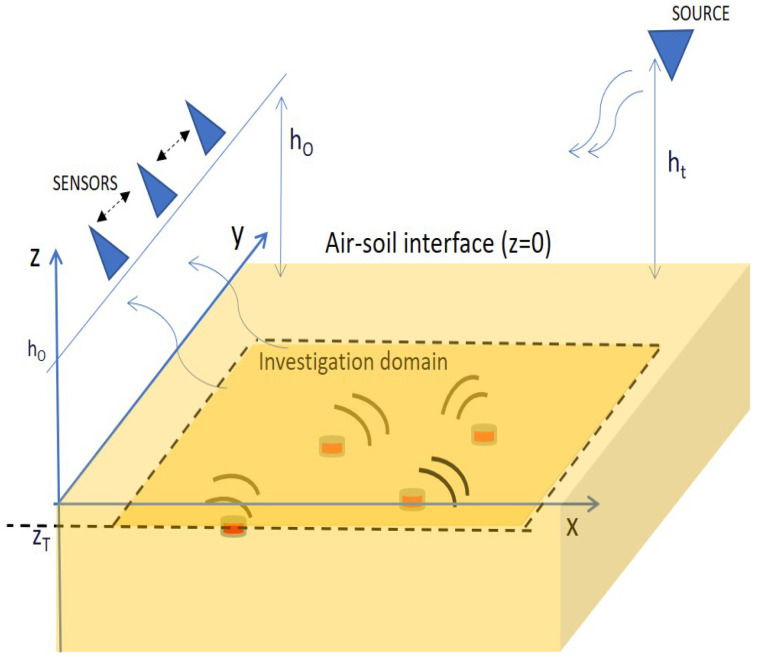
Reference system: the air/soil interface is at z=0, the investigation plane at $z_{T}$.

The scattering scene is probed by a single source located in the upper half-space at some stand-off distance from the air/soil interface, $h_{t}$, whereas the field scattered by the buried targets is collected over a set of sensors still located on the air side and all at the same height $h_{O}$. Accordingly, the spatial measurement positions lay over a plane; say $r_{n}=\left(x_{n},y_{n},h_{O}\right)$, $n=1,2,\ldots ,N_{O}$, with their positions, $N_{O}$ being the number of spatial measurements. Figure 1 shows a pictorial view of the scattering configuration along with the adopted reference frame.

After the scene is illuminated by the incident field, the scattered field arises. Since all the targets are located in the half-space $z<z_{T}$, by invoking the equivalence theorem [21], the scattered field can be considered as being radiated by equivalent surface currents supported over the plane at $z=z_{T}$. In particular, by filling the half-space $z<z_{T}$ with a perfect electric conductor, only the magnetic surface current survives. Such a current can be expressed as

(1) $$J_{m}\left(r;k\right)=J_{eq}\left(x,y;k\right)\delta \left(z-z_{T}\right).$$

Note that the magnetic equivalent current depends on the scattering scene and on the incident field. As such, it depends on the working frequency, which is indeed highlighted in (1) in terms of the wavenumber *k*. In particular, if $z_{T}=0$ (i.e., just at the air/soil interface), the current in (1) radiates in free space, and hence the scattered field (in the upper half-space z>0) can be written as

(2) $$E_{S}\left(r;k\right)=\int \nabla_{r}g\left(r,r^{\prime };k\right)\times J_{m}\left(r^{\prime };k\right)dr^{\prime }=\int_{D}\underline{G}\left(r,r^{\prime };k\right)\cdot J_{eq}\left(r^{\prime };k\right)dr_{t}^{\prime },$$

where $r^{\prime }=r_{t}^{\prime }+z_{T}\hat{z}$ is the source point, with $r_{t}^{\prime }=\left(x^{\prime },y^{\prime }\right)$, r is the field point and

(3) $$g\left(r,r^{\prime };k\right)=-\frac{e^{-jk|r-r^{\prime }|}}{4\pi |r-r^{\prime }|},$$

is the free-space 3D scalar Green function. $\underline{G}\left(r,r^{\prime };k\right)$ is the magnetic to electric dyadic Green function, whose expression is given as

(4) $$\underline{G}\left(r,r^{\prime };k\right)=\left[\begin{array}{ccc} 0 & -\frac{\partial g}{\partial z}\left(r,r^{\prime };k\right) & \frac{\partial g}{\partial y}\left(r,r^{\prime };k\right)\\ \frac{\partial g}{\partial z}\left(r,r^{\prime };k\right) & 0 & -\frac{\partial g}{\partial x}\left(r,r^{\prime };k\right)\\ -\frac{\partial g}{\partial y}\left(r,r^{\prime };k\right) & \frac{\partial g}{\partial x}\left(r,r^{\prime };k\right) & 0 \end{array}\right].$$

It must be remarked that, to be rigorous, surface integration in (2) should run over the entire plane z=0. However, since the targets are very close to the air/soil interface (or in general to the reference plane $z_{T}$), it can reasonably be assumed that the current support is very similar to the target’s cross section. Hence, *D* is chosen according to the size of the spatial region to be investigated. Again, in (2), we considered the free-space Green function. This is correct for $z_{T}=0$. When this is not the case, because the reconstruction at a different depths is required, we will still use the same Green function. Indeed, using the free-space Green function avoids dealing with the computation of the Green function pertaining to a layered background medium. What is more, the layered medium Green function requires the knowledge of the electromagnetic features (dielectric permittivity and conductivity) of the soil, which are in general not available. Herein, such background medium electromagnetic parameters are assumed not to be known. By contrast, using the free-space Green function leads to the targets appearing more deeply located, because soil is electromagnetically denser than air. However, this is not a serious drawback if the targets of interest are shallowly buried.

The magnetic surface current $J_{eq}$ has no component along $\hat{z}$. Accordingly, (2) particularizes as

(5) $$E_{S}\left(r;k\right)=\int_{D}\left[\begin{array}{cc} 0 & -\frac{\partial g}{\partial z}\left(r,r^{\prime };k\right)\\ \frac{\partial g}{\partial z}\left(r,r^{\prime };k\right) & 0\\ -\frac{\partial g}{\partial y}\left(r,r^{\prime };k\right) & \frac{\partial g}{\partial x}\left(r,r^{\prime };k\right) \end{array}\right]\cdot J_{eq}\left(r_{t}^{\prime };k\right)dr_{t}^{\prime }.$$

It is seen that $E_{Sx}$ is solely linked to $J_{eqy}$ and $E_{Sy}$ to $J_{eqx}$. Therefore, if one collects separately such field components, then the inverse problem in (5) splits in two identical scalar inverse problems from which one can reconstruct the two source components independently. Then, these reconstructions can be combined as in [20]. However, in general, this is not the case, even in view of the receiving antenna response. More precisely, what one can actually measure is the antenna output voltage. Therefore, in place of (5), the following equation should be considered

(6) $$V\left(r;k\right)=T\left(E_{S}\right)\left(r;k\right),$$

where *V* is the voltage data, and T is a linear operator schematizing the antenna response. Eventually, this is the scattering model from which to start in order to perform the current reconstructions. A few details concerning the reconstruction algorithm along with some further simplifications to achieve such a task are provided in the next section.

## 3. Reconstruction Algorithm

According to (6), the magnetic current components cannot in general be separately reconstructed. Moreover, the antenna response should be known and put into the model. It would be useful to avoid both these issues. Indeed, looking for simultaneously both the source components means to deal with a doubled number of unknowns. Furthermore, antenna response must be measured/known in advance.

As to the first question, if the receiving antenna is linearly polarized, for example, in the x−z plane, then its plane-wave spectrum vector belongs to the same plane. Accordingly, the main contribution to the voltage is due to $J_{eqy}$, which is the one that contributes to $E_{Sx}$. Similar considerations apply if the other source component is considered. Therefore, we make the problem scalar by approximating (6) as

(7) $$V\left(r_{n};k\right)=\int_{D}H\left(r_{n},r^{\prime };k\right)J_{eqy}\left(r_{t}^{\prime };k\right)dr_{t}^{\prime },$$

where $r_{n}$ are the measurement positions (antenna phase center) and $H(r_{n},r^{\prime };k)$ is the kernel of the integral operator in (7), which is actually the approximation of the composition between the antenna response operator and the propagator, once the contribution due to $J_{eqx}$ has been neglected. However, the antenna response is still there.

It is noted that both the data and the unknown depend on the frequency *k*. Hence, employing all the available multiple-frequency data to perform the reconstruction is not formally allowed. On the one hand, single-frequency reconstructions do not allow one to estimate targets’ depths. This is a minor drawback for shallowly buried targets. Moreover, depth at which reconstruction is achieved can be changed. On the other hand, at a single frequency, the antenna response basically introduces a complex weight, which is the same for each measurement position. This means that it does not affect source localization, which is instead related to the phase term that depends on $r_{n}-r^{\prime }$. Hence, since we are mainly interested in the detection and the localization of the targets (i.e., we do not aim at quantitative reconstructions, even in view of the other approximations), antenna response is neglected in (7) while achieving single-frequency reconstructions. Note that this would not be the case for multi-frequency reconstructions, since the complex weight in general changes with frequency.

In order to perform the reconstruction, the presented model (at a given frequency $k_{l}$) is discretized by representing the unknown current component $J_{eqy}$ as a truncated Fourier series, that is,

(8) $$J_{eqy}\left(x,y;k_{l}\right)=\sum_{n=-N}^{N}\sum_{m=-M}^{M}I_{mn}\exp\left[-j\pi \left(\frac{mx}{x_{M}}+\frac{ny}{y_{M}}\right)\right].$$

where $I_{nm}$ are the Fourier coefficients and the exponentials represent the two-dimensional Fourier spatial harmonics over extent of the domain of investigation. Accordingly, the unknowns of the problem now become the expansion coefficients $I_{nm}$. The choice of *N* and *M* is linked to the so-called number of degrees of freedom of the problem, reflects the ill-posedness of the inverse problem at hand, and depends on the operating frequency as well as the investigation and the observation domain extensions [19,20]. In general, however, this discretization scheme allows one reduce to a large extent the number of unknowns, as compared to a pixel based representation. The corresponding discrete model is then obtained as

(9) $$V(k_{l})=H\cdot I,$$

where $V\left(k_{l}\right)\in C^{N_{O}}$ is the data vector at the *l*-th frequency, $H\in C^{N_{O}\times \left(2N+1\right)\left(2M+1\right)}$ is the matrix version of the scattering operator, and $I\in C^{\left(2N+1)(2M+1\right)}$ is the (vectorized) expansion coefficient matrix.

Equation (9) is inverted for I by a standard Truncated Singular Value Decomposition (TSVD) [25] of the relevant matrix operator. This allows to counteract the ill-posedness of the problem and to obtain a stable reconstruction.

Once the coefficients $I_{nm}$ have been recovered, the corresponding equivalent current $J_{eqy}\left(x,y;k_{l}\right)$ is computed by means of (8). Then, the support of such an equivalent surface current is provided by the image in the x,y investigation domain,

(10) $$I\left(x,y;k_{l}\right)=\left|J_{eqy}\left(x,y;k_{l}\right)\right|.$$

In order to limit the system complexity, the number of spatial data must be reduced. This in general can lead to a reconstruction that is corrupted by aliasing artifacts that are difficult to distinguish from the actual current. To cope with this drawback, a simple strategy based on the combination of single-frequency reconstructions has been introduced in [20]. In more detail, suppose that $N_{k}$ is the number of adopted frequencies; then the final reconstruction is obtained as

(11) $$I\left(x,y\right)=\Pi_{l=1}^{N_{k}}I\left(x,y;k_{l}\right).$$

The very basic idea behind (11) is that aliasing artifacts change positions with the working frequency, whereas the actual source reconstruction does not. Therefore, (11) tends to mitigate all those peaks in the reconstruction that do not overlap (or overlap only partially) while the frequency changes. A criterion for the choice of the frequencies is provided in [20].

In sum, the algorithm presents the following steps:

1. Fix one frequency value;
2. Compute the scattering matrix model H;
3. Compute the SVD of H;
4. Fix a regularizing threshold for the normalized singular values of H (in the following experimental results, 20 dB is used) and compute the unknown vector I via a TSVD inversion by retaining the data projection over the singular vectors corresponding to the singular values above the threshold;
5. Calculate $I(x,y;k_{l})$ by (8) and (10);
6. Repeat from point 1 by changing the frequency value;
7. Compute I(x,y) from (11).

## 4. Experimental Results

In this section, we check the proposed algorithm against some experimental measurements collected in a semi-controlled scattering scenario. In particular, we first describe the test site and then show some reconstructions aiming to highlight the role of the number of spatial measurements and of the employed frequencies.

### 4.1. Test Site

The test site consisted of a tank full of sand of about 3.5 m (length) 2.5 m (width) and 1.5 m (depth) in size. The tank was placed in the open air so that the sand appeared wet, apart from the very surface layer, which was dried by sun. The electromagnetic features of the sand were unknown and wer not estimated for detection purposes.

**Figure 2:**
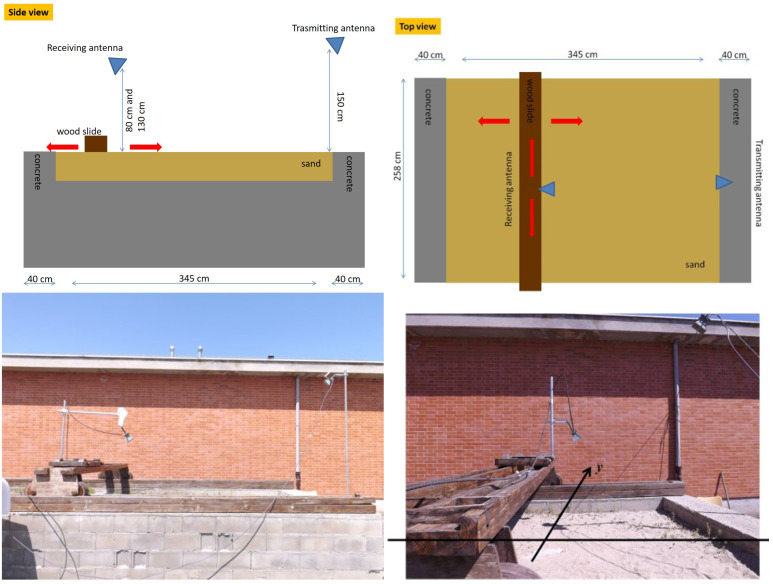
Schematic view of the measurement configuration (**top**) and photos of the test site (**bottom**).

The transmitting antenna was a horn positioned at a $h_{t}=1.5$ m height from the sand floor and located at one of the end sides of the tank. It was tilted to point to the spatial region under investigation. The receiving antenna was still a horn and was located on the other side of the tank. In particular, it was mounted on a wooden slide that allowed it to synthesize a planar measurement aperture at a fixed height from the air/sand interface (in the following examples $h_{O}=80$ cm or $h_{O}=130$ cm). Furthermore, the receiving antenna was tilted toward the investigated spatial region and was linearly polarized. Figure 2 shows a schematic view of the measurement configuration along with some pictures of the test site. As a target, a metallic rectangular plate 17.5 cm × 48 cm in size is considered.

A vector network analyzer was connected to the antennas by means of coaxial cables. Standard calibration at the end of each channel was performed at the beginning of each measurement stage in order to avoid mismatch between VNA and cables. Data have been acquired in the frequency band [2–9] GHz (201 equispaced frequencies) for each different position of the receiving antenna.

We performed measurements under two different conditions: *flat* and *rough* air/sand interface. Flatness was obtained by manually using a shovel for smoothing the sand floor. Of course, the obtained sand surface, though smooth, was far from “ideally” flat. Roughness interface was instead obtained by turning over the sand. For such scenarios, we took measurements with and without targets (background measurements) for comparison purposes.

It is worth remarking that the measurement scenario actually contained many features that are not accounted for in the scattering model used to develop the detection algorithm. For example, though antennas were tilted towards the scattering scene, they still presented a direct link, which implies direct coupling between the receiving and the transmitting antennas. Furthermore, because of the finite dimensions of the tank, the two-half-space medium assumption clearly does not correspond to the actual background medium. This entails that the received signal actually consisted of different contributions besides the one expected from the targets. In addition, note that, even though the medium was a perfect two-layered medium with a flat separation interface, the air/sand interface reflection always superimposes the target signals. Nonetheless, in the following reconstructions, we did not mitigate such unwanted contributions by data pre-processing.

### 4.2. Detection Results for Flat Air/Soil Interface

We start by considering the case of flat air/sand interface in the sense clarified above.

For this case, we collected data over a grid of 5×7 positions. To this end, the receiving antenna scanned the measurement aperture with a spatial step (along both the *x* and *y* directions) of 20 cm at the height and $h_{O}=80$ cm.

**Figure 3:**
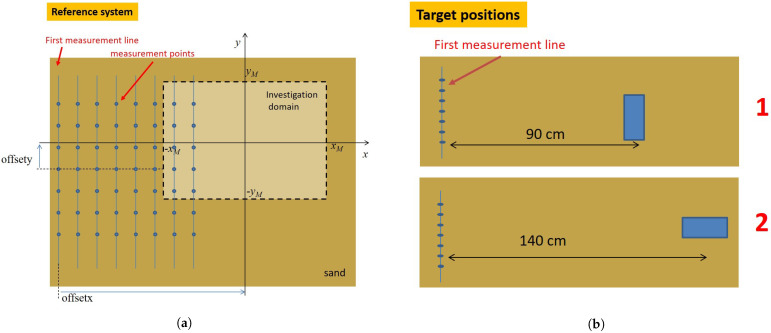
(**a**) Investigation domain and measurement points; the reference system is centred on the investigation domain. (**b**) Different positions of the target with respect to the first (numbered from the left) measurement line.

The investigation domain is a rectangle of 160 cm along the *x* and 100 cm along the *y* direction, and it is located at $z_{T}=0$. We also introduce the two parameters *offsetx* and *offsety*, which indicate the displacement, along the *x* and *y* directions, respectively, of the center of the investigation domain with respect to the central point of the first measurement line (see Figure 3a for the reference system). Basically, after acquiring the data, changing the investigation domain center location (by varying the offset parameters) entails looking for the targets in different spatial regions. Accordingly, the same target will appear at different relative positions. This can be considered as a way to check reconstructions’ consistency and stability. A detection can be considered successful if the target localization point moves coherently with the change of the investigation domain center position.

It must be remarked that the number of employed spatial data is already below the one required if the field has to be properly spatially sampled (see [20]). This, of course, limits the highest frequency that can be used in the reconstructions. Indeed, we tested the inversion algorithm against different bands inside the available one of [2–9] GHz. As expected, when dealing with higher frequencies, even by our approach, detection is impaired because the reconstruction results were crowded by a number of artifacts. Hence, we ended up processing data collected only within the band [2–4] GHz. In particular, in such a band, $N_{k}=11$ frequencies were considered in order to be as close as possible to frequency selection criterion provided in [20].

Finally, the target was located as shown in Figure 3b and roughly buried 2 cm below the air/sand interface.

**Figure 4:**
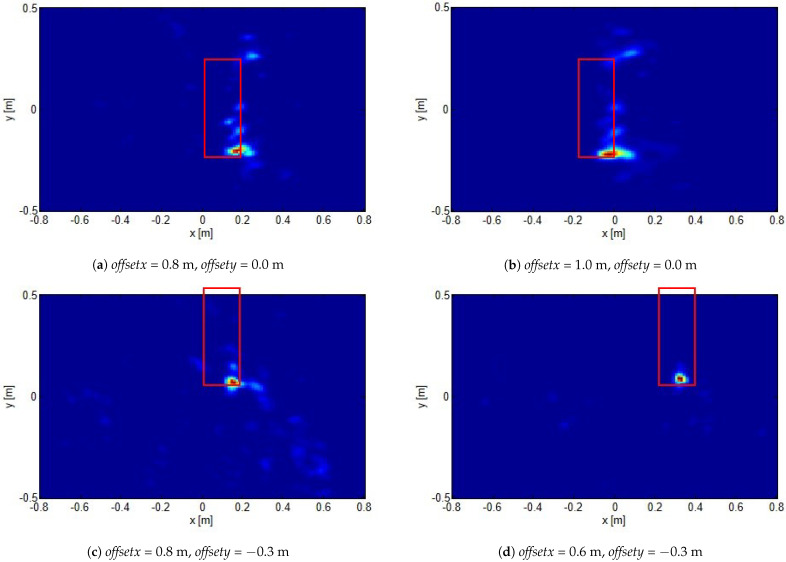
Normalized reconstructions for target located at position 1 shown in Figure 3b for different investigation domain offsets (reported in the figures). The red rectangles show the actual target positions.

In particular, Figure 4 reports the reconstructions for a target located as in position 1 sketched in Figure 3b, for different investigation domain center offsets. As can be seen, a good detection was achieved with no significant artifacts corrupting the reconstructions. What is more, the reconstructed spot moved coherently with the investigation domain displacement.

Some comments are in order here.

Firstly, we remark that the inversion algorithm does not aim at providing the shape of the targets, but rather, it is intended for target detection. This is mainly due to the strategy we adopted to combine the different single frequency reconstructions. Indeed, the proposed multiplicative combination highlighted in (11) allows us to mitigate aliasing artifacts but at the same time tends to enhance the strongest part of the reconstructions so that targets actually appear as hot spots.

**Figure 5:**
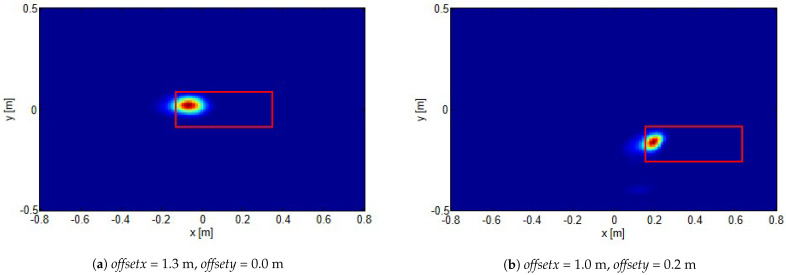
Normalized reconstructions for target located at position 2 shown in Figure 3b for different investigation domain offsets (reported in the figures). The red rectangles show the actual target positions.

**Figure 6:**
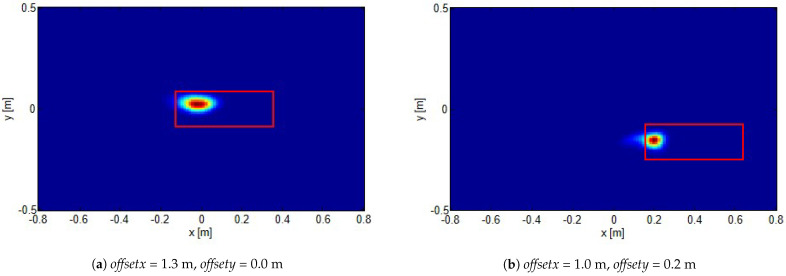
The same as in Figure 5 but incident field polarized orthogonal with respect to the previous case.

Secondly, as we mentioned above, we did not process data to counteract the direct link (from the transmitting antenna to the receiving one) or the air/sand reflection. However, reconstructions do not suffer from such spurious signals. This can be justified by observing that we have performed the reconstruction over a given spatial region: the investigation domain. Hence, the direct link should appear located outside such a region. As to the air/sand interface reflection, it actually enters in the investigation domain. However, it is not localized as the target contribution and tends to be spread over the whole investigation domain. The multiplicative combination strategy hence also enhances the target reconstruction against such a contribution. In Figure 5 and Figure 6, reconstructions corresponding to the target (still approximately buried at 2 cm) located as in position 2 (see Figure 3b) are reported. In particular, while Figure 5 has been obtained using the same source polarization as in the previous case, in Figure 6, the transmitting antenna has been rotated 90${}^{\circ }$ so that the scene is illuminated by an orthogonal polarization. As can be seen, in this case as well, the target is clearly detected, regardless of the transmitting antenna features (in this case polarization). Actually, according to the formulation and related approximations presented in Section 2 and under Equation (7), what matters is that the equivalent source contributes to the polarization and that the receiving antenna is sensitive. Hence, even by changing the transmitting antenna polarization, it is expected that the method works as long as the previous statement holds true. This is basically what happened in Figure 6. In other words, unless the equivalent current has rigorously no component to which the receiving antenna is sensitive, the method is expected to work.

### 4.3. Detection Results for Rough Air/Sand Surface

We now turn to consider the case in which the air/sand interface was not smoothed. In this case, we consider data collected over a grid of 8×7 positions with the same spatial step as above but at a height $h_{O}=130$ cm. Note that the spatial data are slightly greater than the previous case but still under-sampled [20].

**Figure 7:**
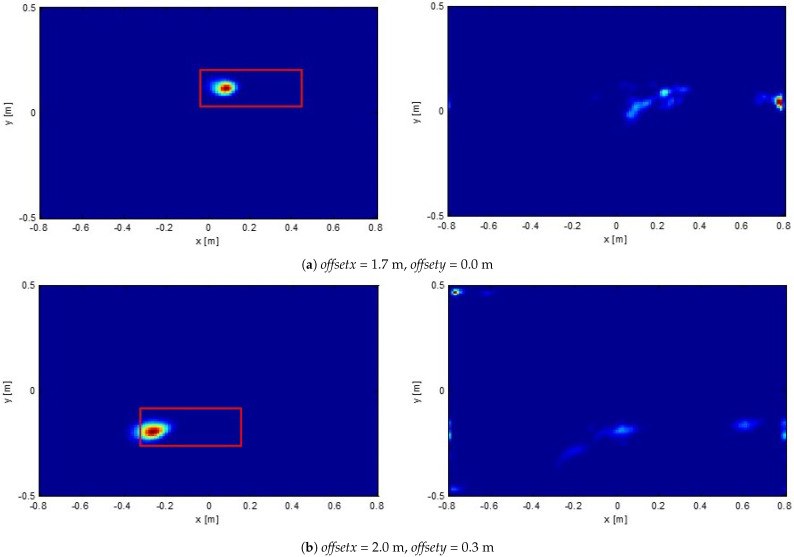
Normalized reconstructions of a shallowly buried targets for two different investigation domain offsets along with the actual target location denoted as red rectangles (figures un the left column). Normalized reconstructions background medium data, i.e., in absence of target, (figures on the right column).

First, we show the reconstruction of a shallowly buried target (the target depth and type are the same as above) by employing all the available frequencies. These results are reported in the left column of Figure 7. As can be appreciated, the target is clearly detected and the related hot spot indicator changes position accordingly to the investigation domain center offset. On the same figure (right column), instead, we report the reconstructions obtained by processing background data, i.e., in absence of the target. Differently from the target case, now the reconstructions do not exhibit a clear hot spot. Moreover, the reconstruction changes as the investigation domain center offset varies. This confirms the previous discussion that processing air/soil interface reflection does not return a focused hot spot. Furthermore, the reconstruction corresponding to this contribution is in general different when the investigated spatial region changes. This is in particularly true here because roughness entails that the air/soil interface has different spatial details. Eventually, these results suggest a possible strategy to recognize actual targets against surface clutter. Indeed, comparing images obtained using different investigation domain offsets, the actual targets are those ones for which the reconstructions “move” coherently with the change in the investigation domain center.

**Figure 8:**
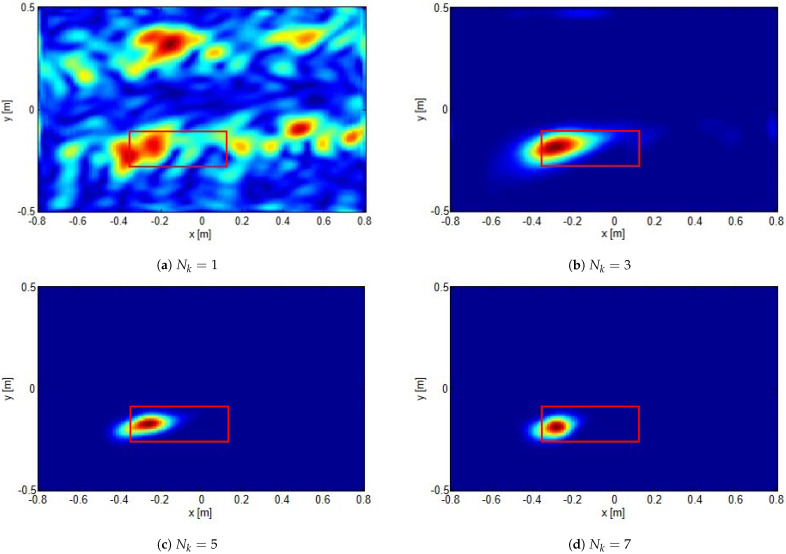
The same case as in Figure 7 with *offsetx* = 2.0 m, *offsety* = 0.3 m. Comparison of reconstructions obtained by employing different number of frequencies $N_{k}$.

In Figure 8, as well as in Figure 7, by keeping fixed the investigation domain with *offsetx* = 2.0 m, *offsety* = 0.3 m is considered. However, different numbers of frequencies are employed. As can be seen, when the number of frequencies is increased, the aliasing artifacts actually tend to disappear and the hot spots narrows. This is, of course, expected and perfectly consistent with the theoretical arguments discussed above.

The simple proposed strategy for reducing spatial data hence works very well. To further check this procedure, we consider an even more challenging case by reducing the spatial data employed to achieve the reconstructions. In more detail, we collected data over a 4×4 measurement grid, with twice the spatial step, that is, 40 cm. The height of the measurement aperture was still $h_{O}=130$ cm, and the frequencies were taken within the same band as above. Note that in this case the number of spatial data is even lower than the ones used in the first example reported at the beginning of this section.

**Figure 9:**
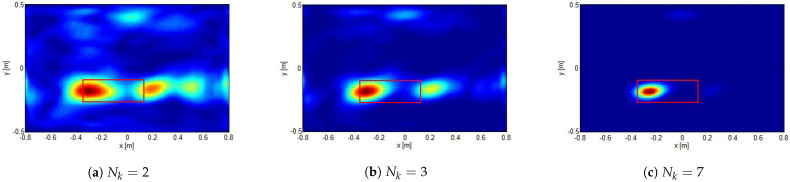
Normalized reconstructions of a shallowly buried target corresponding to the case reported in Figure 7, *offsetx* = 2.0 m, *offsety* = 0.3 m but with only 4 × 4 measurement grid collected with a spatial step of 40 cm. The actual target location is denoted as red rectangles. Comparison of reconstructions obtained for different values of $N_{k}$.

It can be seen in Figure 9 that even under this more challenging situation where the spatial data have been reduced further, the proposed method works very well in detecting and localizing the target and in counteracting aliasing artifacts, though the number of frequencies is actually low as well.

**Figure 10:**
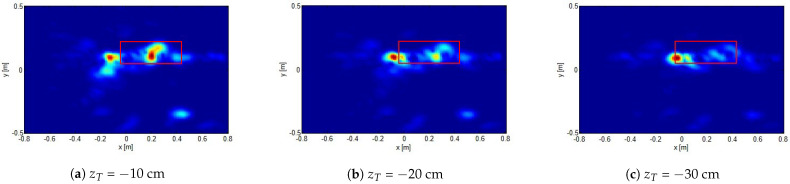
Normalized reconstructions of a target approximately buried 10 cm below the air/sand interface. Data have been collected over 4 × 4 measurement grid with a spatial step of 40 cm and *offsetx* = 1.7 m, *offsety* = 0.0 m. $z_{T}=7$ at different depths.

As a final example, we consider the case the target is buried more deeply. In particular, in this case, the plate target is buried approximately 10 cm below the air/sand interface. Figure 10 shows the reconstructions obtained by considering the image plane at different depths. In all the examples, $N_{k}=7$ frequencies (which were shown to be enough for mitigating aliasing artifacts) have been employed, and the same spatial grid measurement as in Figure 9 is considered. These results show that the proposed method is still effective in detecting the target. Moreover, as discussed above, changing the depth at which reconstruction is achieved allows one to get information about the target depth as the one for which the reconstruction is more focused. Of course, since the soil permittivity is not known (and hence not accounted for in the reconstruction algorithm), the estimated target depth does not coincide with the actual one. In particular, since the relative dielectric permittivity of a wet sand likely stands between 4 and 9, the “apparent” target depth can range from 20 cm to 30 cm, which is perfectly consistent with the result shown in Figure 10.

A brief comment is in order about the actual location of the reconstructed spots that, in most of the figures above, appear focused at the edges of the true shape. As a matter of fact, one can expect that the detected hot spot should roughly appear in correspondence with the target center. Indeed, this circumstance has been observed in [19,20], where synthetic data were employed. However, in those cases, we have considered a reflection-mode configuration (i.e., the TX (plane wave incidence) and the RXs were on the same side), the investigation domain was centered with respect to the measurement aperture, and the target was precisely parallel to the reconstruction plane. For the present case, because of the experimental set up, of course, the target position and its orientation are not precisely known; uncertainty is a few cm. In addition, the configuration is quite different since a transmission-mode set up is under consideration and the measurement aperture is actually side-looking the investigation plane. All these aspects could have contributed to the obtained results. However, we believe that such reconstructions are most likely due to the illumination, which is not uniform across the target and depends on the relative position between the TX and the target itself. In order to obtain a clear understanding of this effect, one should go through the study of the equivalent current behavior. This can be numerically achieved and is beyond the aim of the paper.

## 5. Conclusions

In this paper, we experimentally validated a method for detecting and localizing shallowly buried targets from under-sampled multi-frequency data. The method relies on two main ingredients: a suitable scattering model and a simple procedure to process multi-frequency under-sampled data. The scattering model is based on the equivalence theorem, which allows one to cast the detection as the reconstruction of equivalent sources supported over a reference plane. This way, detection becomes a 2D problem. Furthermore, the incident field is embodied into the unknown equivalent sources. Therefore, coherence between the TXs and Rxs is not necessary when single-frequency data are employed. Reconstructions obtained at different frequencies are then suitably combined. This allows one to mitigate aliasing artifacts and hence to achieve the reconstructions with a very reduced number of spatial measurements.

Experimental results confirm the feasibility of the method even under a rather complex scattering scenario, such as the one addressed herein. Indeed, it is shown that the targets can be detected by using very few spatial measurement points and a number of properly selected frequencies. In addition, the results confirm the robustness of the method against the reflection occurring at the air/soil interface, even when this exhibits some degree of roughness.

The obtained results can be meant as a proof of concept. However, they encourage the application of the proposed measurement configuration and of the related inversion algorithm to more challenging scenarios, where many targets may be present inside a non-homogeneous terrain and the sensors may be deployed at a larger stand-off distance.

## Data Availability

No new data were created or analyzed in this study. Data sharing is not applicable to this article.
